# Setting Agenda for Medical Education Research in Pakistan

**DOI:** 10.12669/pjms.37.3.3603

**Published:** 2021

**Authors:** Sarah Ali, Ahsan Sethi

**Affiliations:** 1 Sarah Ali Assistant Registrar, Pakistan Medical and Dental Council, Islamabad, Pakistan; 2 Ahsan Sethi Assistant Professor, Institute of Health Professions Education and Research, Khyber Medical University, Peshawar, Pakistan

**Keywords:** Health Professions, Medical Education, Priority, Research

## Abstract

**Objective:**

To justify the allocation of human and financial resources, this study aims to identify multiple stakeholders’ views of medical education research priorities in Pakistan for the next five years.

**Methods:**

This two-stage exploratory mixed-method study was conducted from Jan 2018 to Jun 2019. A purposively selected sample of 250 faculty members, research supervisors, postgraduate students, undergraduate students and policymakers actively involved in improving Medical Education were included. In Stage-I: An exploratory open-ended questionnaire asking about Medical Education Research (MER) priorities in Pakistan for the next five years was emailed. Data were thematically analyzed to identify MER areas. In Stage-II: Another questionnaire was developed based on MER areas. The participants were asked to rate their importance on a scale of one to five. Descriptive statistics were calculated using SPSS.v.24.

**Results:**

In Stage-I, 140 participants and in Stage-II, 130 participants from different stakeholder groups responded. We identified 20 research priorities grouped under eight themes: curriculum organization, content, delivery, assessment, workplace, students, faculty and educational management. Top three research priorities were identifying needs and developing effective provisions for continuous professional development of the faculty, improving assessment and communication skills.

**Conclusion:**

The study identified top MER priority areas as continuous professional development, assessment and communication skills. Some areas unique to the current study include admissions, fostering critical thinking, Islamic values in professionalism and ethics. The study provides evidence-base for decision-making about allocating time and funds for MER in Pakistan.

## INTRODUCTION

Medical education has emerged notably as a specialty in many countries abroad, but in Pakistan it has been recognized relatively recently. The interest in medical education can be evaluated by the increasing number of postgraduate programmes in this field worldwide (7 to 126+) and Pakistan (2 to 8), over the last two decades.^1^ Such programmes improve knowledge and encourage transformational changes in educational practices with development as teacher, learner leader and researcher.^1^,^2^ These roles of a medical educator have been recognized in the literature.^3^

Medical education research aims at developing an understanding of teaching and learning by studying interactions, interventions and phenomena, thus providing evidence-base to medical educators and policymakers.^4^ Therefore, it has a profound impact on the healthcare system.^5^ The Higher Education Commission (HEC) Pakistan has developed policies and programmes to encourage research among faculty members in the country.^6^ Likewise, the statutory body governing medical education in Pakistan has made the teaching of research mandatory in the undergraduate and postgraduate curriculum. Research in the respective specialty has been formally linked with promotions of the faculty.

In 2010, the HEC created Offices of Research Innovation & Commercialization (ORIC) in every Higher Education Institution for ‘transforming Pakistani universities to drive high impact innovation, applied research and entrepreneurship’.^7^ Therefore, the medical universities must ensure that their research effectively contribute towards improvement in the health and wellbeing of their community. The World Health Organization also emphasized on social accountability in medical institutions: ‘the obligation to direct their education, research and service activities towards addressing the priority health concerns of the community, region, and/or nation they have a mandate to serve’.^8^

Few countries (New Zealand,^9^ Canada,^10^ Scotland,^11^ Iran^12^ and Eastern Mediterranean Region^13^) have previously conducted priority-setting exercises for Medical Education Research (MER). Such studies help ensure that MER contributes most effectively towards improvement in existing undergraduate, postgraduate and continuing medical education system. These may also increase ownership of the priorities as interests of relevant people are considered and thus facilitate educational reforms.^14^ Pakistan, is a developing country with limited number of trained medical educationist and research funds,^15^ which mandates evidence-informed decision making. Our healthcare system, values, culture and hence the MER priorities may differ substantially from those identified in other countries. Hence, the current study aims to identify multiple stakeholders’ views of Medical Education Research (MER) priorities in Pakistan for the next five years. The study is timely and will help ensure utilization of resources wisely to maximize research productivity in medical education and healthcare.

## METHODS

This two-stage exploratory mixed-method study was conducted from Jan 2018 to Jun 2019. Ethical approval was granted by Ethics Review Committee, Islamic International Medical College, Rawalpindi (RIPHAH/IIMC/ERC/17/0241 Dated:04-07-2017).

### Questionnaire:

In Stage-I: An exploratory open-ended qualitative questionnaire was developed asking the participants about three Medical Education Research (MER) priorities in Pakistan for the next five years. In Stage-II: Another questionnaire was developed based on MER areas (subthemes/themes) identified in Stage-I asking the participants to rate the importance of these areas on a Likert scale of one (not important) to five (very important) and add new priority areas if they perceived them as absent. The questionnaires were checked for understanding and accessibility.

### Data Collection:

Participants included purposively selected faculty members, research supervisors, postgraduate students, undergraduate students (International Federation of Medical Students’ Associations), Standing Committee on Medical Education) and policy makers (statutory body members), which are actively involved in improving ‘Medical Education’ in Pakistan. An information sheet and questionnaire were shared through email with 250 participants across the country. Two reminders were sent to encourage participation.

### Data Analysis:

For qualitative data, the researchers independently read each response and developed a thematic framework of in-vivo codes. The codes were then categorized into subthemes and themes.^16^ Through constant comparison method the subthemes/themes were continuously refined. Quantitative data were analysed using SPSS.v.24. Frequencies and percentages were calculated for demographics. Likert scale ratings of importance for each of the 20 subthemes were computed as medians and interquartile ranges (IQRs). The ratings given to each subtheme by all the participants were summated to identify the total rank scores and the overall rankings.

## RESULTS

In Stage-I, 140 participants and in Stage-II, 130 participants from diverse stakeholder groups responded. Most of the respondents had postgraduate qualifications in medical education. Majority were from Punjab and Khyber Pakhtunkhwa province. (Table-I).

**Table-I:** Participant Characteristics from Stage-I and Stage-II.

Characteristics		Undergraduate Medical Students		MHPE Graduates (Faculty members)		Medical Education Supervisors/Policymakers	

		Stage-I n (%)	Stage-II n (%)	Stage-I n (%)	Stage-II n (%)	Stage-I n (%)	Stage-II n (%)
Mean Age (Years)		21.45±1.36	38.20±9.86	41.33±8.73	42.59±9.07	48.29±9.84	40.95±10.46
Gender	Male	6 (30.0)	11 (55.0)	43 (54.4)	44 (60.3)	25 (61.0)	18 (48.6)
	Female	14 (70.0)	9 (45.0)	36 (45.6)	29 (39.7)	16 (39.0)	19 (51.4)
Area	Islamabad	2 (10.0)	2 (10.0)	14 (17.7)	13 (17.8)	10 (24.4)	7 (18.9)
	Punjab	10 (50.0)	10 (50.0)	40 (50.6)	25 (34.2)	12 (29.3)	20 (54.1)
	Sindh	2 (10.0)	3 (15.0)	4 (5.1)	10 (13.7)	13 (31.7)	4 (10.8)
	KPK	1 (5.0)	3 (15.0)	20 (25.3)	23 (31.5)	5 (12.2)	5 (13.5)
	Baluchistan	5 (25.0)	2 (10.0)	1 (1.3)	2 (2.7)	1 (2.4)	1 (2.7)

We identified 20 research priorities grouped under eight themes: curriculum organisation, content, delivery, assessment, workplace, students, faculty and educational management. Top research priorities were identifying needs and developing effective provisions for continuous professional development of the faculty, designing valid and reliable assessments and also ensuring their quality and standardization, enhancing health professionals’ communication amongst each other and the patients or their relatives (Table-II).

**Table-II:** Medical Education Research (MER) priorities in Pakistan.

Theme	Subtheme	Definition	Median (IQR)	Total Rank Score (overall ranking)
Curriculum organization	Integration of basic and clinical sciences	This refers to improving understanding of the horizontal and vertical integration of disciplines around body systems, organs or themes in the curriculum.	4 (4-5)	575 (12)
	Community orientated medical education	This refers to the inclusion and prioritization of curriculum content based on the evolving needs of the community.	5 (4-5)	587 (6)
	Defining core curricula	This refers to defining standardized minimum core curriculum contents nationwide.	4 (4-5)	581 (10)
Curriculum content	Improving communication skills	It is important for health professional to learn to communicate effectively with each other and the patients or their relatives.	5 (4-5)	603 (3)
	Professionalism and ethics with consideration to Islamic values	This means defining and teaching medical professionalism and ethics in line with Islamic values.	5 (4-5)	596 (4)
	Use of technology and innovation in medical education	It refers to the incorporation of new technology and innovations in medical education.	4 (4-5)	574 (14)
Curriculum delivery	Fostering reflective and critical thinking	It refers to enhancing metacognitive, reflective and lateral thinking among students for clinical decision-making and solving complex problems	4 (4-5)	582 (9)
	Engaging near peers in teaching	This means encouraging a process of teaching and learning amongst peers i.e. Peer Assisted Learning	4 (4-5)	514 (20)
	Improving assessment practices	This refers to designing valid and reliable assessments and also ensuring their quality and standardization.	5 (4-5)	607 (2)
Assessment and feedback	Delivering effective feedback	It means to understand effective ways of giving and receiving feedback.	4 (4-5)	582 (8)
	Development of leadership	To understand the development of leadership skills in healthcare context.	4 (4-5)	577 (11)
Workplace	Teamwork and interprofessional learning	This refers to the understanding of learning with from and about other healthcare professionals for working together as an effective and efficient healthcare team.	5 (4-5)	585 (7)
	Workplace-based learning	This encompasses the learning of clinical skills in the workplace	4 (4-5)	566 (16)
	Understanding role modelling	This mean understanding the learning of norms, values and practices through role modelling.	4 (4-5)	536 (19)
Students	Counselling and mentoring	It refers to the role of counselling and mentoring in helping learners resolve problems and set career-related goals.	4 (4-5)	561 (17)
	Admission and promotion of medical students	It means development of admission and promotion policies and processes for students in medical colleges and universities.	4 (4-5)	575 (13)
Faculty	Continuous Professional Development	This relates with identifying needs and developing effective provisions for continuous professional development of the faculty.	5 (5-5)	616 (1)
	Recruitment, appraisal and promotion criteria	This refers to developing appropriate job description, recruitment process, workload models and promotion regulations for the faculty	4 (4-5)	573 (15)
Educational management	Total quality management	It encompasses quality assurance, quality control and accreditation	5 (4-5)	595 (5)
	Advancing inclusion and diversity	It refers to policies and practices that support diversity and inclusion initiatives in medical education.	4 (4-5)	538 (18)

## DISCUSSION

This is the first study on setting agenda of medical education research (MER) in Pakistan for next five years. We identified eight themes of medical education research as priorities: curriculum organisation, content, delivery, assessment, workplace, students, faculty and educational management. There is a need to organize the research work around these priorities identified. The resources may be diverted towards researching these areas instead of reliance on research patterns defined by individual academicians based on interests. The outcomes of such prioritization exercises are usually relevant to the context in which it is carried out. In terms of MER areas there are more similarities than differences among our findings and those in other countries.^9^-^13^ Therefore, we believe that these results may be relevant across the international arena. We recommend similar priority-setting exercises to researchers from other specialties (dental and nursing etc) and in other countries in order to set their MER agendas. All these efforts may contribute towards developing an international MER agenda.

There are differences in terms of the rankings of MER areas identified in the current study and those in other studies. In this study, the top research priority areas were related to effective provisions for continuous professional development, assessments and enhancing communication amongst each other and the patients or their relatives. In Scotland,^11^ the highly ranked area was balancing intersecting clinical and educational identities,^17^ while faculty development was ranked #18, which received the highest ranking in our study. Likewise, assessment and communication skills were ranked much lower. These findings are in line with our educational landscape, which is currently evolving with a move towards curriculum integration and more student-centered learning strategies, which demands designing effective means for training of the faculty. There is also a difference in the interpretation of these medical education areas. For example, in Pakistan and Iran^12^ research is needed over the integration of basic and clinical sciences at undergraduate level, whereas, in Scotland,^11^ it refers to the integration of undergraduate and postgraduate education. It is pertinent to mention here that like any other research priorities, those in medical education might change over the time and therefore would need continuous assessment. For example, the recent COVID-19 pandemic has resulted in diversion of resources and research in facilitating online teaching/learning through various medical education adaptions worldwide.^18^ Probably, if this study is repeated today, the use of technology and innovation in medical education (rank#14 in Pakistan) would have ranked much higher as a priority worldwide.

Some areas unique to the current study include admission and promotion of medical students, advancing inclusion and diversity in medical education, role modelling, fostering critical thinking, Islamic values in professionalism and ethics. A recent study on predictive ability of the medical students’ admissions criteria suggested a weak correlation with grades in the professional examinations.^19^ They suggested assessment of non-cognitive attributes in the admission process. Similarly, other studies^20^,^21^ also suggested teaching Islamic perspective of medical professionalism that consists of ‘faith (Iman), consciousness (Taqwat), best character (Ahsan al Akhlaq), excellent performance (Itqaan al’Amal), strife toward perfection (Ihsan), responsibility (Amanat), and self-accountability (Muhasabat Alnafs)’. These unique areas reflect areas that need attention in our context. The MER should help develop indigenous and contextually relevant guidelines, norms and standards, rather than passively following those guidelines that do not represent the Pakistani cultural context.^22^ The regulatory bodies of medical education in Pakistan must ensure appropriate funding and protected time for research in medical institutions.

### Limitations of the study:

In the current study, we selected a purposive sample of faculty members, research supervisors, postgraduate students, undergraduate students and policy makers, who are actively involved in improving ‘Medical Education’ across institutions and provinces of Pakistan. Moreover, our participants mostly belonged to Punjab, Khyber Pakhtunkhwa and Sindh with fewer participants from Baluchistan province. These participants may or may not be representative of MER stakeholders in the country.

## CONCLUSION

The study identified top medical education research priority areas as continuous professional development, assessment and communication skills. There are more similarities than differences between our findings and those from other countries. Some areas unique to the current study include admission and promotion of medical students, advancing inclusion and diversity in medical education, role modelling, fostering critical thinking, Islamic values in professionalism and ethics. The study provides evidence for researchers, funding bodies, health institutions and policymakers to base decisions on allocation of time and funds for MER in Pakistan.

### Authors’ Contribution:

**SA & AS:** Conceived the idea and designed the study.

**SA:** Was involved in data collection.

Both the authors performed data analysis and contributed towards writing the manuscript and approving the final version, are responsible for integrity of the study.
